# Fracture Resistance and Stress Distribution Pattern of Different Posts-Core Systems in Immature Teeth: An In Vitro Study and 3D Finite Element Analysis

**DOI:** 10.1155/2022/2610812

**Published:** 2022-02-09

**Authors:** Shahnaz Khadar, Kishor Sapkale, Pravinkumar G. Patil, Sayed Abrar, Manoj Ramugade, Febel Huda

**Affiliations:** ^1^Department of Conservative Dentistry and Endodontics, Government Dental College and Hospital, Mumbai, MUHS Nashik, Maharashtra, India; ^2^Department of Prosthodontics, Division of Restorative Dentistry, School of Dentistry, International Medical University, Kuala Lumpur, Malaysia; ^3^Cochin Implant Centre, Kalamaserry, Ernakulam, India

## Abstract

**Introduction:**

Restoration of immature teeth with open apices and thin dentinal walls with conventional post systems remains a challenge. The purpose of this study was to evaluate the fracture resistance of simulated immature teeth restored with different intraradicular posts and assess their stress distribution pattern using 3D Finite Element Analysis (FEA).

**Methods:**

Fracture strength testing using universal testing machine was carried out in simulated immature teeth restored with different intraradicular posts grouped as follows: Group A: teeth not restored with posts served as control group; Group B: Cast metal post (CMP); Group C: Customized Composite Post (CCP); Group D: Fiber post (FP). Four 3D FEA models of the above groups were created using CATIA^TM^ software and analyzed for stress distribution using ANSYS^TM^. The results of fracture strength testing and FEA were correlated. Multiple group comparisons were analyzed by one-way ANOVA followed by Tukey HSD post hoc test.

**Results:**

The CMP exhibited highest fracture resistance (336.43 N) but resulted in root fractures. The CCP exhibited lower fracture resistance (240.90 N) and favorable stress distribution as compared to CMP. The FP and control group exhibited lower fracture resistance values of 182.69 N and 130.46 N, respectively. The results of 3D FEA demonstrated higher stress concentration in model comprising metallic post and core.

**Conclusions:**

Teeth restored with cast metal posts and cores exhibited maximum fracture resistance followed by the customized composite posts, the fiber posts, and the control group. The cast metal posts indicated higher von Mises stresses concentrated in the radicular region; however, the customized composite posts, the fiber posts, and the control group demonstrated stress concentration in the coronal region.

## 1. Introduction

To be categorized as successful, endodontic treatment should render the affected tooth free of signs and symptoms and enable adequate clinical function. However, endodontic treatment often increases the fracture susceptibility of teeth, which can be attributed to operative procedures and moisture loss [1]. Thus, it is crucial to provide a structurally and functionally sound restoration following endodontic treatment. The treatment planning becomes highly critical in case of immature teeth with open apices. Loss of vitality of a developing tooth due to trauma or extensive caries results in thin, divergent dentinal walls of root canal with open apex. Such teeth are weak particularly in cervical areas of root, thereby increasing fracture susceptibility even to normal functional stresses [2]. The general protocol for management of immature teeth with diseased or necrotic pulp involves root canal treatment including apexogenesis or apexification procedures followed by protective permanent coronal restorations [3]. For over five decades, calcium hydroxide apexification remained the mainstay for management of teeth with incompletely formed apices. This treatment protocol, however, necessitates multiple appointments and may cause further weakening of the root [4]. Presently advocated method of single step apexification using MTA provides several advantages, which include shorter treatment time, excellent biocompatibility, and good apical seal [5]. Revascularization is yet another alternative for management of immature teeth. It aids in regeneration of pulpal tissue and eventually promotes continued root development and apical closure [6]. However, there is little evidence providing established success rates and predictability of the outcome, despite promising results shown by several case reports [7].

Immature teeth generally present with weakened dentinal walls especially in the cervical area. The restoration of immature incisors with the use of flowable fiber-reinforced post-core composites displayed promising performance in a matter of fatigue-resistance and survival [8]. Martins et al. [9] systematically reviewed ten studies regarding the evidences about the failure rates of endodontically treated teeth restored with intraradicular metal posts or fiber posts and found out no difference for failure rates between metal posts and fiber posts [9]. Various posts have been used to rehabilitate structurally compromised immature teeth. Use of prefabricated posts in wide flared canals in immature teeth presents a challenge due to mismatch in their diameters. Improper fit and excessively thick layer of resin cement may cause air bubble formation predisposing to debonding [10]. The lack of adaptability could be overcome with the use of cast metal posts. However, inherent high modulus of elasticity of the metallic posts might be deleterious to the structural integrity of the remaining tooth structure. Boudrias et al. described a technique that advocates relining of the fiber post with composite to ensure better adaptability to the root canal [11]. It has been proposed that these customized composite posts, also known as anatomic posts, may have better mechanical and physical properties. The stress distribution pattern in these posts is similar to fiber posts and is evenly dissipated throughout the root canal, and the chances of unfavorable fractures are reduced. [12]

Various destructive and nondestructive tests can be used to assess the physical properties and mechanical behavior of teeth and restorations. In the last decade, Finite Element Analysis (FEA) has become a popular method for analyzing dental biomechanics. It allows simulation of the geometry and loading conditions of the structures to be analyzed [13]. The aim of this study was to assess the fracture resistance of simulated immature teeth restored with different posts and the stress distribution pattern on 3D Finite Element Models.

## 2. Materials and Methods

Eighty extracted human single rooted maxillary central incisors extracted for periodontal reasons were used as study samples. The samples were stored in distilled water at room temperature until further use for not more than 8 weeks [14]. The teeth were screened preoperatively. Single rooted maxillary central incisor teeth with straight roots were included in the study. Teeth with fractures and visible cracks and morphological variations and caries were excluded from the study. Mesiodistal and labiolingual dimensions of all the teeth were measured using Vernier caliper and samples presenting a difference of more than 20% of the mean (Mesiodistal 7 ± 2 mm, labiolingual 6 ± 2 mm) were discarded. [15] Out of the 80 samples, 20 were segregated to constitute Group A-control group.

### 2.1. Preparation of Control Group (Group A)

**Figure 1:**
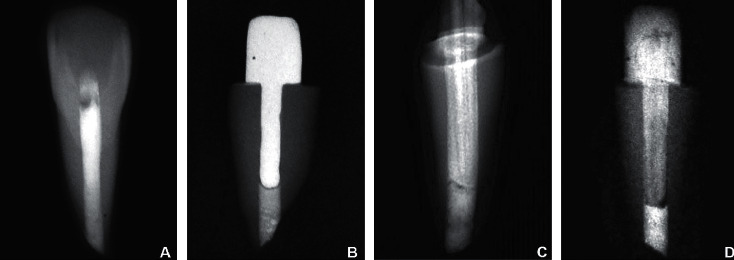
(a) Radiograph indicating tooth prepared with apical plug of MTA and obturation of remaining root embedded in self cure acrylic resin. (b) Radiograph after cementation of cast metal post and core. (c) Radiograph after cementation of customized composite post. (d) Radiograph after cementation of fiber post and core buildup.

Access into the root canals was gained using round diamond point BR41 (MANI INC). The canals were instrumented till #80 K file (MANI INC). Peeso reamers (MANI INC) between #1 to #6 were sequentially introduced in the canal and passed 1 mm beyond the apex to simulate teeth with open apices. The root canals were irrigated using 2 ml of 5% Sodium Hypochlorite (Tripharma) after each instrument, and a final flush with 17% EDTA (Canalarge; Ammdent) was done to remove smear layer. The root canals were finally flushed with distilled water and dried using paper points. Single visit apexification technique was simulated by placing a 4-5 mm thick MTA ANGELUS^®^ apical plug in each tooth. The MTA-carrier was used to place the material apically and condensed using an endodontic plugger. A wet cotton pellet was used to cover the apices of these teeth, and a moist paper point was placed on the coronal side of the MTA plug. The samples were stored at room temperature for 72 hours to allow MTA to set completely [15]. The root canals were obturated using lateral condensation technique with gutta percha and resin sealer (Figure 1(a)). The access openings were sealed using composite resin.

### 2.2. Preparation of Experimental Groups (Groups B, C, and D)

The anatomic crowns of 60 teeth to be used as experimental groups were sectioned perpendicular to the long axis of the tooth using a diamond disc under copious water cooling and ground flat using an abrasive paper, and the length of all the samples was standardized (12 ± 1 mm). After decoronation, round diamond point BR- 41, ISO no. 014 (MANI INC), was used in a high speed airotor hand piece (NSK) to enlarge the access cavity. The canals were instrumented to ISO no. 80 K file. The apices were enlarged to simulate immature teeth, and 4–5 mm of MTA was placed in all the teeth as described in the control group. The teeth were then randomly assigned to three experimental groups, namely, Group B: Cast metal post and core (CMP); Group C: Custom composite post and core (CCP); and Group D: Fiber post and composite core (FP). The details of the sample preparations in each group are as follows:

### 2.3. Group B- CMP

Direct wax pattern was taken using burnout post and inlay wax. The coronal portion was standardized to a height of 5 mm with a step at the palatal surface to be used as a standardized point for load application. The wax patterns were cast using lost wax technique with Nickel Chromium alloy. The posts were cemented using dual cured resin cement (Maxcem Elite; Kerr) following standard procedures (Figure 1(b)).

### 2.4. Group C- CCP

A glass fiber post (Size 3, Hi rem; Overfibers) was conditioned with 37% phosphoric acid gel for 15 seconds, followed by rinsing and drying. The fiber post was coated with silane (Silano; Angelus) for one minute, and the surface was gently air-dried for five seconds. An adhesive was applied and light-cured for 10 seconds. The canal was lubricated with a hydrosoluble gel (KY Jelly; Johnson & Johnson). The fiber post was covered with a nanohybrid composite resin (3M ESPE), and the set was inserted into the canal. This set was removed and replaced twice, and the excess cervical resin composite was removed. The composite resin was light-cured for 20 seconds with the post inside the root canal. The relined fiber post was then removed, and the composite resin was additionally light-cured for 20 seconds on each surface for additional polymerization. The post was cemented using dual cure resin cement using standard procedure (Figure 1(c)). The core was built up with a fiber core and a nanohybrid composite resin through incremental filling. Each increment was light-cured for 20 seconds. A matrix with the same dimensions as the core of the previous group was used for core standardization.

### 2.5. Group D- FP

Size 3 glass fiber posts (Hi rem; Overfibers) with maximum diameter of 1.8 mm with 0.08% conicity were used. The glass fiber posts were conditioned as described in group C. They were cemented to the root canals using dual cure resin cement using standard procedures. Later, the coronal portion of the cemented post and tooth surface was etched and bonded, and the core of standard dimensions was prepared with composite (Figure 1(d)).

### 2.6. Fracture Testing of the Specimens

The samples were perpendicularly embedded in self-curing acrylic resin in identical size cylinders. They were then mounted on a specifically designed inclined jig made of steel and tested in a universal testing machine (ACME). A compressive load was applied 2 mm cervical to the incisal edge on palatal aspect at an angle of 135 degrees to the long axis of the tooth at a crosshead speed of 1 mm/min. Failure threshold was defined as the point at which the loading force reached the maximum value to fracture the root, post, or core. The fracture strength values were recorded in Newton (N), and the location of fracture was also assessed radiographically.

### 2.7. Finite Element Analysis

**Figure 2:**
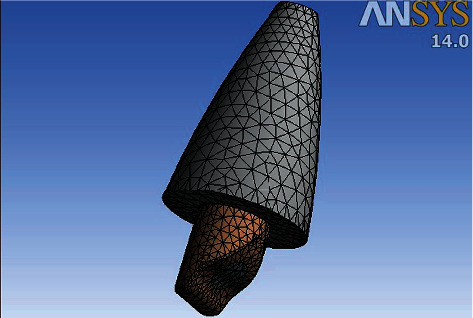
3D finite element model of immature maxillary central incisor.

3D models were created using CATIA^TM^ software based on dimensions of mature maxillary central incisor obtained from literature [16]. The apical diameter was prepared to be 1.60 mm in diameter corresponding to #6 Peeso reamer. The cores were designed to a height of 5 mm with a 2 mm circumferential ferrule (Figure 2). The modulus of elasticity and Poisson's ratio of components of teeth, supporting structures, and restorative materials were obtained from literature [17] as indicated in Table 1. It was assumed that all structures and materials were homogenous, isotropic, and in possession of linear elasticity. The models assessed using ANSYS^TM^ software were grouped as follows: Model 1: endodontically treated and access restored with composite; Model 2: restored with cast metal post and core; Model 3: restored with customized composite post and core; Model 4: restored with fiber post and composite core. Each of these models was subjected to standard loading conditions. The direction of force application was horizontal, vertical, and oblique. For horizontal loading, a load of 10 N was applied at a point midway between the cervico-incisal aspect of the crown. For vertical loading, a load of 100 N was applied at the incisal edge of the crown. For oblique loading, a load of 50 N was applied at a 45-degree angle to the long axis of the tooth [18]. In each of the models, the resultant maximum von Mises stresses (VMS), which represent a combination of tensile, compressive, and shear stresses, can be used for predicting the risk of fractures. VMS is a measure of the distortion energy density, whereas principal stress measures the maximum compressive or tensile stress that will be present under specified applied load. Various investigators have reported that tensile stresses or von Mises stresses can be used as indicators of restorative failures [19, 20]. Color coding was used to depict the stress distribution pattern where blue indicates areas of least stress concentration and red depicts areas of highest stress concentration.

The stresses observed during oblique loading in these models were correlated with the location of fracture in teeth subjected to fracture strength test. Fracture resistance of above groups under static loading was determined by using universal testing machine, and the results obtained were tabulated and statistically analyzed. Mean and standard deviation were determined for all the three groups. Multiple group comparisons were analyzed by one-way ANOVA followed by using multiple comparisons Tukey HSD post hoc test. The *P* < 0.001 was considered for statistical significance.

## 3. Results

### 3.1. Fracture testing results (Tables 2–4)

**Figure 3:**
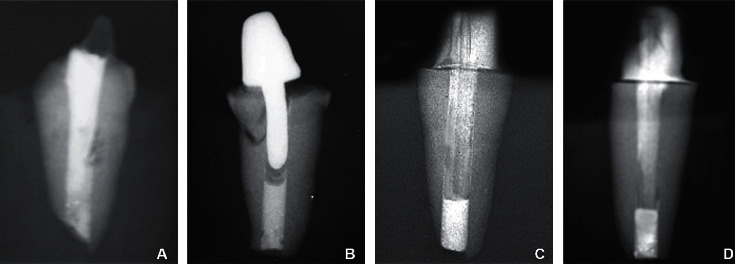
(a) Radiograph indicating failure pattern of: control group. (b) Cast metal post and core. (c) Customized composite post and core. (d) Fiber post and composite core.

The Group A-control group exhibited the lowest mean fracture resistance of 130.46 N. All the samples presented with fracture of the coronal tooth structure (Figure 3(a)). The Group B-simulated immature teeth restored with cast metal post and core exhibited highest mean resistance of 336.43 N. All the samples of Group B presented with fractures of the root (Figure 3(b)). Group C-simulated immature teeth restored with customized composite post and core exhibited mean fracture resistance of 240.90 N. The pattern of failure observed in all the samples of this group was fracture of the composite core (Figure 3(c)). Group D-simulated immature teeth restored with fiber post and composite core exhibited mean fracture resistance of 182.69 N. The pattern of failure observed was core fracture (85%) and core fracture with debonding of the post and or/core (15%) (Figure 3(d)). The differences in the fracture resistance values between Group A, Group B, Group C, and Group D were statistically significant with *P* < 0.001.

### 3.2. 3D Finite Element Analysis results (Table 5) (Figures 4–7)

Maximum von Mises stresses were concentrated in the cervical third of the root in model 2, and the cores of models 1, 3, and 4. In Model 1, overall stress under vertical, oblique, and horizontal loading was 30.194 MPa, 12.698 MPa, and 6.341 MPa, respectively. In Model 2, overall stress under vertical, oblique, and horizontal loading was 39.87 MPa, 19.37 MPa, and 9.514 MPa, respectively. In Model 3, overall stress under vertical, oblique, and horizontal loading was 36.205 MPa, 14.91 MPa, and 6.871 MPa, respectively. In Model 4, overall stress under vertical, oblique, and horizontal loading was 36.157 MPa, 14.63 MPa, and 6.766 MPa, respectively. The overall von Mises stresses were least in Model 1, compared to Model 2, Model 3, and Model 4. Higher von Mises stresses were observed in Model 2, compared to the other models. The mode of failure of teeth restored with different posts subjected to fracture strength testing can be correlated to the areas of stress concentration observed in 3D Finite Element Analysis. Maximum fractures occurred in the core region of Groups A, C, and D, which is in accordance with areas of stress concentration observed in FEA. The model representing teeth restored with cast metal post and core demonstrated maximum stress concentration in the cervical and middle thirds of the root, which is consistent with the fracture of roots in all the samples of Group B.

## 4. Discussion

For decades, cast metal post and cores have been the modality of choice for restoring endodontically treated teeth with compromised coronal structure, with a considerably high clinical success rate [21]. It has been a favorite among skilled clinicians, because it offers precise fit, excellent retention, and high strength. However, the inherent high modulus of elasticity, unfavorable stress concentration, and resultant catastrophic fractures has led the way for development of newer materials and techniques [22]. In recent years, there has been an upsurge in the use of prefabricated fiber posts, due to its perceived advantages especially low modulus of elasticity and esthetics. However, the use of a prefabricated fiber post in case of wide and flared canals, as is the case in immature teeth, is challenging due to the difference in the diameter of the post and root canal. Improper fit of the post results in a thick layer of resin cement with entrapment of air bubbles especially in the coronal third, predisposing it to debonding [23]. To address this concern, the concept of relining the fiber post with composite emerged, which enables close adaptation of the post to the root canal walls.

In this study, the posts in all the groups were cemented with self-adhesive dual cure resin cement following standard procedures. Studies have elucidated that the adhesion of self-adhesive resin cements to root dentin is comparable to that of conventional resin cements used with etch and rinse adhesive systems and is suitable for cementation of intraradicular posts [24, 25]. A dual cure cement was used to allow for completion of chemical reaction in deeper areas where light would be unable to reach. The samples were embedded perpendicularly in self-cure acrylic resin in identical sized cylinders. The effect of periodontium was not simulated in the present study. Using wax or silicon for simulating periodontium may cause root movement during loading. Also, the elasticity of these materials is different than that of periodontal ligament and may not be ideally representative of clinical conditions [26]. However, the periodontal ligament was simulated in the finite element model to avoid creating exaggerated stresses on the outer surfaces of the root [21]. Also, the cores were not restored with crowns in the experimental groups, and the compressive load was applied directly on the palatal surface of cores, as in various similar studies [27–29]. Placement of a crown could alter the distribution and transmission of stresses in the post root complex, obscuring the reinforcing effect of the tested modalities. [30]

The control group (Group A) consisting of simulated immature teeth restored with gutta percha without a post exhibited the lowest fracture resistance. This could be attributed to the cohesive strength and modulus of elasticity of gutta percha being too low to reinforce the roots of endodontically treated teeth [31]. Also, enlarging the canals to simulate immature teeth may have caused weakening of the tooth structure [32]. Results of this study indicate that cast metal post has highest fracture resistance. The inherently high modulus of elasticity enables the post to withstand deformation forces generating high stress concentration at interfaces [32, 33]. The teeth restored with customized composite posts (group C) demonstrated intermediate fracture resistance values. The improved mechanical behavior of customized composite posts in comparison to fiber posts can be attributed to close adaptation of posts to canal wall as stated by Macedo et al. [34]. The lower fracture resistance of CCP as compared to CMP could be due to difference in the modulus of elasticity of composite and resin cement as well as the presence of an additional interfaces for bonding between the post-composite and composite-resin cement. An indirect customized composite post could be used as an alternative to overcome this shortcoming [12]. However, considering the normal masticatory loads, which rarely exceed 75–125 N in the natural dentition in the anterior region [35], the fracture resistance values of custom composite posts demonstrated in this study may be considered clinically acceptable. However, these posts have not yet been extensively studied for post retention and cyclic fatigue. The teeth restored with fiber posts demonstrated lower fracture resistance in comparison to the other two experimental groups in this study. The high resistance of the metal post group could be due to the inherent property of the metal. Poorly fitted posts may create levers within the root canal [13], making the post more liable to fracture or debonding as observed in the study.

The results of the 3D-FEA suggest that stress concentration occurs where a nonhomogeneous distribution of stresses is present, such as the interfaces of materials with varying moduli of elasticity. Peak stress values were observed at the point of load application and cervical region in all the models. Model 2, comprising metallic post and core, demonstrated higher stress concentration especially in the radicular region. These results are in concurrence with Adanir and Belli [13] and Al-Omiri et al. [10] who observed higher intracanal stress values in teeth restored with metallic posts. It was further observed in this study that both custom composite posts and fiber posts demonstrated even distribution of stresses in radicular dentin. This can be attributed to similarity in modulus of elasticity and the possible formation of a monoblock, which enables even distribution of stresses along the tooth structure. [21]

The results of this study suggest that immature or flared teeth can be more favorably restored with customized composite posts as they demonstrated higher fracture resistance than teeth restored with fiber posts and favorable stress distribution pattern than cast metal posts. However, FEA is a software driven, virtual computational analysis, which may not be able to duplicate actual clinical situation. In FEA, it is assumed that all the materials tested are isotropic and homogenous and do not reproduce the heterogeneity of the biologic tissues and dental materials. But the comparative nature of this study makes these assumptions acceptable as the aim was to compare the stress distribution among the models and not to quantify stresses in each model [36]. Another limitation of this study is that a single static load was used for testing the fracture resistance. The forces generated by masticatory and parafunctional activities occur in several directions instead of the single direction as in compressive fracture strength tests [37]. Thermocycling and fatigue loading would have more closely simulated clinical scenario [38]. Adequate discretion is required while extrapolating the results of the present study to clinical context. The loads resulting in fractures of tested groups are quite high in comparison to the physiological loads generated in the oral cavity. Most fractures of the post and core units occur after several years and are rarely related to acute overload but result from fatigue failure [23].

The results obtained from the present in vitro study cannot be directly extended to clinical situation; however, they do provide reproducible and reliable means for comparing and testing the fracture resistance of immature teeth restored with different post systems by both fracture strength testing and 3D FEA method. Most of the fractures were observed in the cervical third of post/core/tooth junction, which can be correlated to the results of FEA, which showed maximum stress concentration in the cervical third region. The crowns were not simulated in the 3D FEA models to avoid the influence of the different crown occlusal anatomy. Further studies can be advocated with inclusion of the real clinical situation simulation. More clinical trials are required to discern the advantages of customized composite posts. Further research is required to substantiate the results of the present study.

## 5. Conclusions

Within the limitations of this in vitro study, the following conclusions were drawn:

1. Teeth restored with cast metal posts and cores exhibited maximum fracture resistance followed by the customized composite posts, the fiber posts, and the control group
2. The cast metal posts indicated higher von Mises stresses concentrated in the radicular region; however, the customized composite posts, the fiber posts, and the control group demonstrated stress concentration in the coronal region

## Figures and Tables

**Figure 4:**
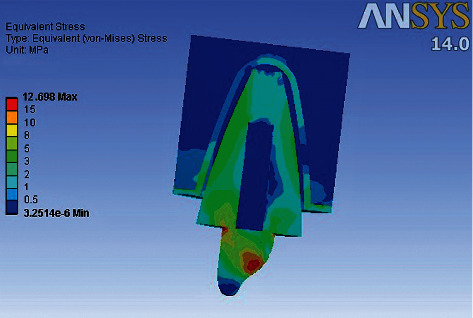
Areas of stress concentration in Model 1 on oblique loading.

**Figure 5:**
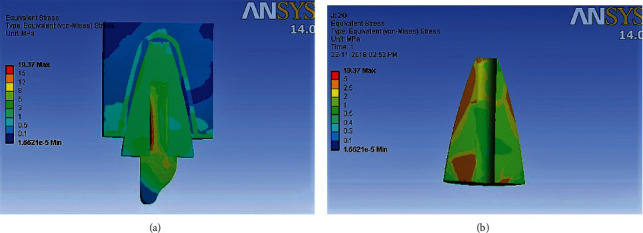
(a) Areas of stress concentration in Model 2 on oblique loading. (b) Areas of Stress concentration in Model 2 in root cross section.

**Figure 6:**
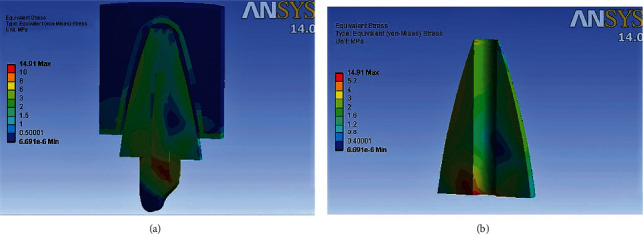
(a) Areas of stress concentration in Model 3 on oblique loading. (b) Areas of stress concentration in Model 3 in root cross section.

**Figure 7:**
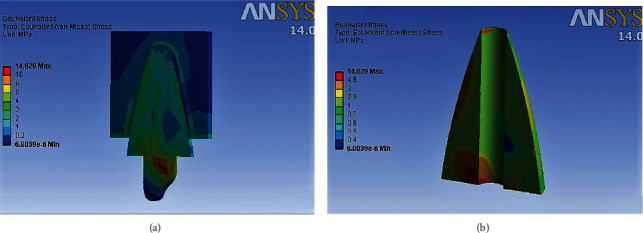
(a) Areas of stress concentration in Model 4 on oblique loading. (b) Areas of stress concentration in Model 4 in root cross section.

**Table 1:** Modulus of Elasticity and Poisson's ratio of different materials.

	Young's modulus (GPa)	Poison's ratio

Enamel	84.1	0.33
Dentin	18.6	032
Periodontal ligament	6.89 × 10^−5^	0.45
Cortical bone	13.7	0.30
Cancellous bone	1.37	0.30
Composite resin	11.3	0.30
Resin cement	9.5	0.30
MTA	11.7	0.31
Metal (Ni Cr)	200	0.33
Gutta percha	0.69	0.45

**Table 2:** Comparison of fracture resistance under static loading amongst four groups using one-way ANOVA.

	*N*	Mean(*N*)	Std. deviation	Std. error	95% confidence interval for mean		Minimum	Maximum	*P* value
					Lower bound	Upper bound			
Control	20	130.46	42.43	9.49	110.60	150.32	74.50	215.07	<0.001
Cast metal post and core	20	336.43	62.72	14.03	307.07	365.78	259.85	536.43	
Custom composite post and core	20	240.90	33.61	7.52	225.17	256.63	184.65	308.60	
Fiber post and composite core	20	182.69	22.70	5.08	172.06	193.31	142.10	233.80	

**Table 3:** Inter group comparison between Control and Experimental groups using Tukey HSD post hoc test.

GROUP (I)		Mean difference (I-J)	Std. error	95% confidence interval		*P* value
				Lower bound	Upper bound	
Control	Cast metal post and core	−205.967	13.58348	−241.6481	−170.2859	0.001
	Custom composite post and core	−110.4445	13.58348	−146.1256	−74.7634	0.001
	Fiber post and composite core	−52.2295	13.58348	−87.9106	−16.5484	0.001

Cast metal post and core	Control	205.967	13.58348	170.2859	241.6481	0.001
	Custom composite post and core	95.5225	13.58348	59.8414	131.2036	0.001
	Fiber post and composite core	153.7375	13.58348	118.0564	189.4186	0.001
	Control	110.4445	13.58348	74.7634	146.1256	0.001
	Cast metal post and core	−95.5225	13.58348	−131.2036	−59.8414	0.001
	Fiber post and composite core	58.215	13.58348	22.5339	93.8961	0.001

Fiber post and composite core	Control	52.2295	13.58348	16.5484	87.9106	0.001
	Cast metal post and core	−153.7375	13.58348	−189.4186	−118.0564	0.001
	Custom composite post and core	−58.215	13.58348	−93.8961	−22.5339	0.001

**Table 4:** Failure pattern observed for experimental groups.

Failure mode	Group 1 (*N* = 20)	Group 2 (*N* = 20)	Group 3 (*N* = 20)	Group 4 (*N* = 20)
Fracture of post and/or core	—	—	20 (100%)	17 (85%)
Debonding at post/core/tooth interface	—	—	—	3 (15%)
Root fracture	—	20 (100%)	—	—
Fracture of coronal tooth structure	20 (100%)			

**Table 5:** Stress distribution pattern in finite element models.

	Model 1			Model 2			Model 3			Model 4		
STRESS (MPa)	Ver	Obl	Hor	Ver	Obl	Hor	Ver	Obl	Hor	Ver	Obl	Hor

Cortical bone	5.15	3.8	1.1	5.3	3.7	1.15	4.8	3.6	1.1	5	3.65	1.12
Cancellous bone	1.25	1.15	0.28	1.31	1.2	0.3	1.26	1	0.28	1.25	1.05	0.3
PDL	1.5	1.9	0.4	1.52	1.6	0.42	1.4	1.6	0.38	1.5	1.62	0.4
Dentine root	11	5	2.2	11.5	5.5	1.75	9.5	5.2	1.7	9.6	4.8	1.8
Gutta percha	0.13	0.13	0.021	NA	NA	NA	NA	NA	NA	NA	NA	NA
Ni-Cr post and core	NA	NA	NA	33	15.3	9.5	NA	NA	NA	NA	NA	NA
Fiber post	NA	NA	NA	NA	NA	NA	26	10.5	3.5	25.5	10.8	3.4
Composite in core	9.8	15	5.8	NA	NA	NA	5.5	5.1	1.5	NA	NA	NA
Composite core	NA	NA	NA	NA	NA	NA	30	10.5	4.2	31	10.8	4.5
Dentine core	22.5	10	6.1	NA	NA	NA	NA	NA	NA	NA	NA	NA

Note: Ver- Vertical loading, Obl- Oblique loading, Hor- Horizontal loading.

## Data Availability

The data used to support the findings of this study are available from the corresponding author upon request based on institutional rules and regulations.
